# Phylogenetic Distribution of Phenotypic Traits in *Bacillus thuringiensis* Determined by Multilocus Sequence Analysis

**DOI:** 10.1371/journal.pone.0066061

**Published:** 2013-06-10

**Authors:** Michael B. Blackburn, Phyllis A. W. Martin, Daniel Kuhar, Robert R. Farrar, Dawn E. Gundersen-Rindal

**Affiliations:** Invasive Insect Biocontrol and Behavior Laboratory, Agricultural Research Service, United States Department of Agriculture, Henry A. Wallace Beltsville Agricultural Research Center, Beltsville, Maryland, United States of America; Rockefeller University, United States of America

## Abstract

Diverse isolates from a world-wide collection of *Bacillus thuringiensis* were classified based on phenotypic profiles resulting from six biochemical tests; production of amylase (T), lecithinase (L), urease (U), acid from sucrose (S) and salicin (A), and the hydrolysis of esculin (E). Eighty two isolates representing the 15 most common phenotypic profiles were subjected to phylogenetic analysis by multilocus sequence typing; these were found to be distributed among 19 sequence types, 8 of which were novel. Approximately 70% of the isolates belonged to sequence types corresponding to the classical *B. thuringiensis* varieties *kurstaki* (20 isolates), *finitimus* (15 isolates), *morrisoni* (11 isolates) and *israelensis* (11 isolates). Generally, there was little apparent correlation between phenotypic traits and phylogenetic position, and phenotypic variation was often substantial within a sequence type. Isolates of the sequence type corresponding to *kurstaki* displayed the greatest apparent phenotypic variation with 6 of the 15 phenotypic profiles represented. Despite the phenotypic variation often observed within a given sequence type, certain phenotypes appeared highly correlated with particular sequence types. Isolates with the phenotypic profiles TLUAE and LSAE were found to be exclusively associated with sequence types associated with varieties *kurstaki* and *finitimus*, respectively, and 7 of 8 TS isolates were found to be associated with the *morrisoni* sequence type. Our results suggest that the *B. thuringiensis* varieties *israelensis* and *kurstaki* represent the most abundant varieties of *Bt* in soil.

## Introduction

Early attempts to classify *Bacillus thuringiensis* (*Bt*) relied on classical biochemical tests used to characterize bacteria [1], which were later combined with serotyping of flagellar antigens [2]–[4]. Subsequently, *Bt* isolates were classified for many years based on serotyping alone. Based on these phenotypic characteristics, distinct varieties of *Bt* were assigned names that are still in use to this day, such as *thuringiensis*, *kurstaki*, *israelensis*, *sotto* and *finitimus*, to name a few. More recently, examination of the larger *Bacillus cereus* group by multilocus sequence analysis (MLSA) and Fluorescent Amplified Fragment Length Polymorphism (fAFLP) have yielded considerable insight into the population structure of *Bt* and allied species, and offered a means of classifying them phylogenetically [5]–[10]. These studies have suggested a generally clonal population structure, and revealed three major divisions within the *B. cereus* sensu lato, with *Bt* and *B. cereus* interspersed in two of these clades and mostly *B. cereus*, *B. mycoides*, and *B. weihenstephanensis* occurring in the third. Many serotyped Bt strains have been included in these phylogenetic analyses; these have been found to be dispersed across all three major divisions.

The Invasive Insect Biocontrol and Behavior Laboratory (USDA-ARS, Beltsville, MD, USA) possesses a world-wide collection of *Bt* isolates from soil that have been phenotypically characterized on the basis of classical biochemical tests and crystal morphology [11]. Because the only criterion for inclusion into the collection is a *Bacillus cereus*-like biochemical profile and the production of a parasporal crystal, the collection represents a relatively unbiased sample of *Bt* as it exists in soil. Using the presence or absence of six biochemical traits that have classically been useful in differentiating *Bt*, the collection can be categorized into 64 possible phenotypic profiles. We wished to characterize representatives of common phenotypic profiles by MLSA to determine if there was any correlation between phenotype and phylogenetic position.

## Materials and Methods

Strains were selected from among 3,639 characterized *Bts* isolated from approximately 350 soil samples originating in 34 countries around the world, with 42% of isolates from samples obtained within the United States. The vast majority of these isolates were obtained from soil by acetate selection as described previously [12]. Each isolate was tested for amylase (T), lecithinase (L), urease (U), acid production from sucrose (S) and salicin (A), and hydrolysis of esculin (E). Using these single letter abbreviations, phenotypes were assigned based on positive test results for a trait. Thus, if an isolate produced amylase and lecithinase, but tested negatively for all other traits, the phenotype would be TL. Based on the results of these tests, the relative abundance of all 64 possible phenotypic profiles in the collection was determined in a previous study [11].

Four isolates representing each of 15 common phenotypes were selected for an initial round of phylogenetic analysis using the MLST scheme devised by Priest et al. [5]. In the case of phenotype TL which represents ca. 24% of the collection, and in cases where the initial screening step suggested that a particular phenotype was exclusively associated with one sequence type, additional representatives were analyzed. All strains used and their phenotypic traits are listed in Table S1. Sequences for the multiple loci were amplified for each isolate using primers for the *glpf*, *gmk*, *pta*, *tpi*, *ilvD*, *purh*, and *pycA* loci as designed and described in that earlier study [5], with the exception of a *pta* forward primer (ptaF1 5′- GCGTTTAGCAAAAGAAGAGTTAGTA -3′) developed by our group [13]. For PCR, thirty-five cycles were conducted in a model 9700 thermocycler (Applied Biosystems, Foster City, CA, USA) using 30 sec denaturation at 94°C, 1.5-min annealing at 55°C, and 2-min primer extension (10-min in final cycle) at 72°C. Each gene amplicon was sequenced directly. Products were separated on 1.5% NuSieve agarose gel (FMC, Rockland, ME) in modified 1 X TAE (0.04 M Tris-acetate and 0.1 mM EDTA), and excised for sequencing using ABI BigDye V1.1 (Applied Biosystems, Foster City, CA, USA), using the amplification primers. Cycle sequencing conditions were 35 cycles at 96°C, 10 sec; 50°C, 5 sec; 60°C for 4 min. Automatic sequencing was carried out on an ABI Prism Model 3130xl (Applied Biosystems, Foster City, CA, USA). Amplification of α-urease gene fragments were accomplished with the primer pairs α-ureF1

5′-TGCATTTCATATCCCCACAACA-3′ and α-ureR1 5′-CTGCCGCGATTGTTTCTTTTC-3′. PCR and sequencing parameters for the α-urease gene were identical to those for MLSA except that PCR products were precipitated with 20% PEG 8000 and washed twice with chilled 80% ethanol prior to sequencing. Sequences were edited and assembled using the SeqMan component of DNASTAR (DNASTAR, Inc. Madison, WI, USA). Sequence types were determined by BLAST searches [14] of the PubMLST database [15] for the *Bacillus cereus* group. Phylogenetic analysis of the concatenated sequences for all seven loci from these and all additional sequence types currently in the PubMLST database was conducted and trees were created with MEGA 5 software [16] using maximum likelihood analysis.

## Results and Discussion

Sequence types (ST) associated with the 15 common phenotypic patterns in our sample are presented in Table 1. In all, 19 STs were identified among the isolates, including 8 new STs. Isolate and sequence type data were deposited in the PubMLST *B. cereus* database (http://pubmlst.org/bcereus/). Phylogenetic analyses of the concatenated alleles from all STs in the database, including those from the current study, revealed that most STs occur within the three major divisions that have been reported previously [5]–[7], [9], and also support the additional groups proposed by Guinebretière et al. [8] and Tourasse et al. [10] (Fig. S1). Following the nomenclature suggested by Priest et al. [5], also utilized in the following discussion, these prior studies have shown that Clade 1 includes *B. anthracis* and mammalian pathogens, emetic strains of *B. cereus*, and sporadic examples of *Bt*. Clade 2 includes *B. cereus* and the largest number of *Bt* isolates, and was further subdivided into the lineages Kurstaki, Sotto, Tolworthi and Thuringiensis. Clade 3 comprised primarily *B. cereus*, *B. weihenstephanensis* and *B. mycoides* strains with few *Bts*. Approximately 25% and 75% of the isolates in our sample belonged to STs in Clade 1 and 2, respectively, with a single isolate in Clade 3.

**Table 1:** Phenotypes with associated sequence types.

Phenotype	Percentage of collection*	Sequence types†
TL	23.91	ST-16 (7), ST-240, ST-241, ST-548
TLAE	9.63	ST-8 (3), ST-551
TLS	6.27	ST-16 (2), ST-26, ST-547
TLU	6.22	ST-8 (2), ST-240 (2)
TLA	6.12	ST 8-(3), ST-171
TS	4.82	ST-23 (7), ST-241
TLUAE	4.24	ST-8 (8)
TLE	3.64	ST-8 (2), ST-16, ST-593
TLSA	3.46	ST-10, ST-22, ST-171, ST-241
TLSAE	2.96	ST-171 (5), ST-241 (2), ST-592
LSAE	2.53	ST-171 (8)
TLUA	2.38	ST-8 (2), ST-240, ST-550
Ø	2.08	ST-549 (3), ST-197
T	1.61	ST-239, ST-241, ST-16, ST-23
TSAE	1.03	ST-23 (3), ST-594

* Percentage of the phenotype among 3,639 characterized isolates.

† Values in parentheses represent the number of isolates found (if more than one).

**Figure 1:**
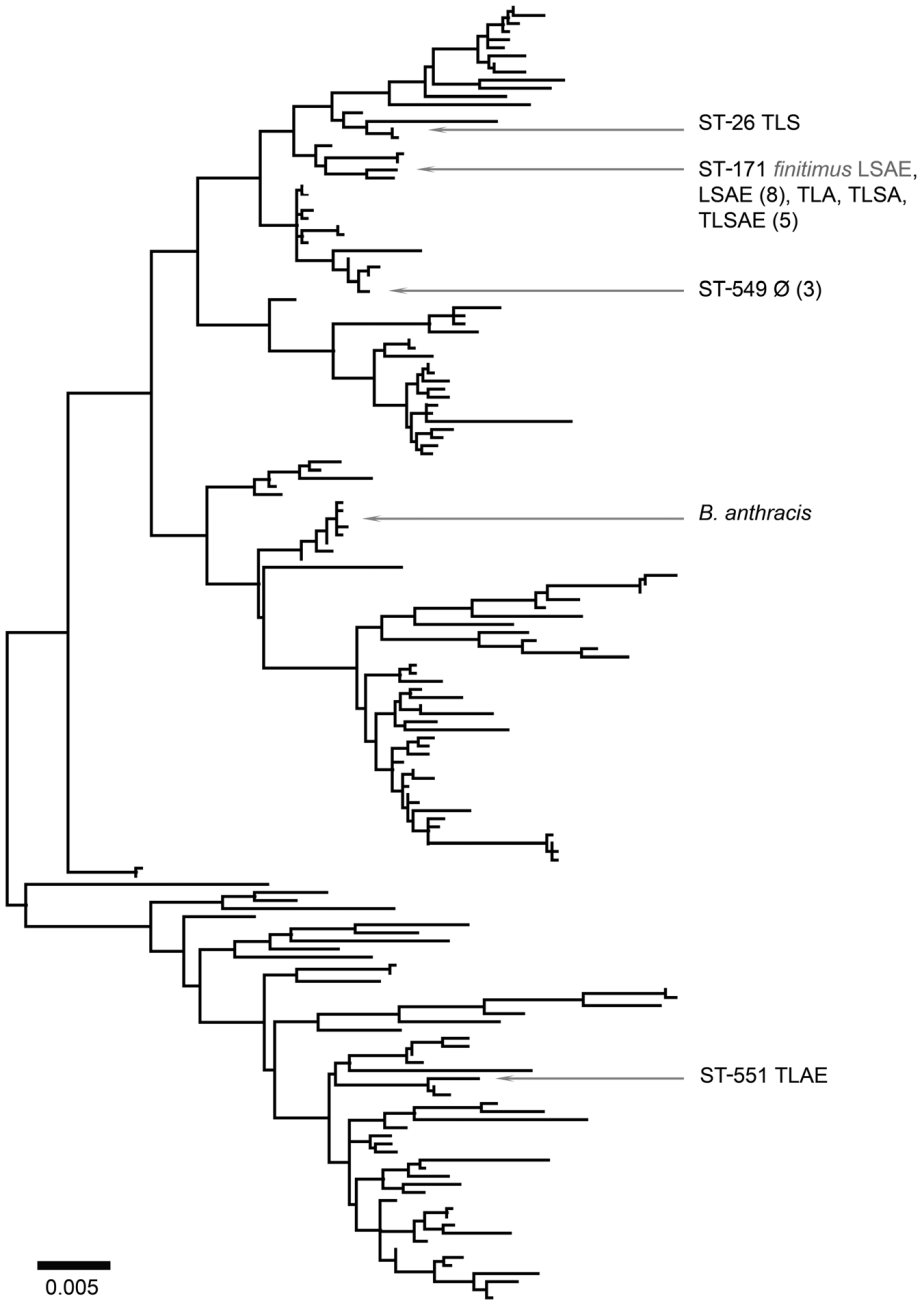
Un-rooted maximum likelihood analysis of Clade 1, with sequence types and corresponding phenotypes found in our sample indicated. Numbers in parentheses indicate the number of isolates with the indicated phenotype observed in the ST. Light text indicates varieties and phenotypes described by de Barjac [3]; *B. anthracis* added for reference.

**Figure 2:**
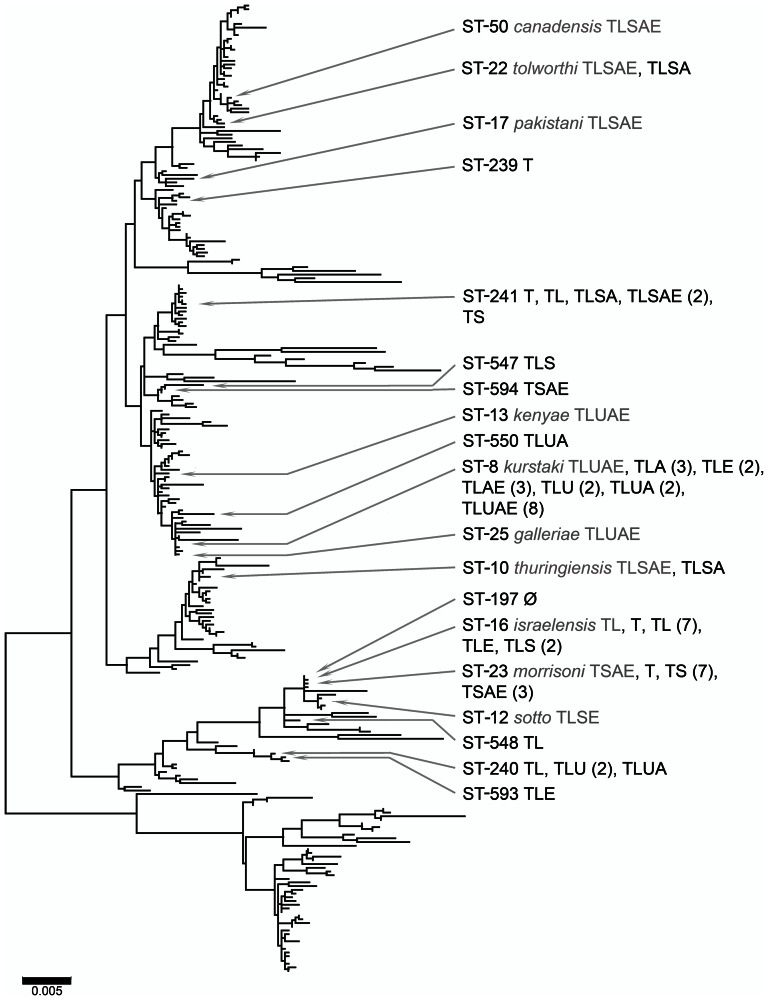
Un-rooted maximum likelihood analysis of Clade 2, with sequence types and corresponding phenotypes found in our sample indicated. Numbers in parentheses indicate the number of isolates with the indicated phenotype observed in the ST. Light text indicates varieties and phenotypes described by de Barjac [3].

The phylogenetic distribution of phenotypic profiles in Clade 1 and Clade 2 are depicted in Figure 1 and Figure 2 respectively. The same trees, with all STs labeled, are presented in Figures S2 and S3 respectively. Examination of the phylograms shows that there were no individual phenotypic traits associated exclusively with any phylogenetic position or group. Not surprisingly, the phenotypic traits that were relatively unusual, such as the *lack of* amylase or lecithinase production, or the *production of* urease, appeared in fewer STs than more frequently encountered traits. Thus, amylase-negative isolates were present primarily in ST-171, lecithinase-negative isolates occurred primarily in ST-23, and urease-positive isolates were almost exclusively associated with ST-8 and ST-240. Even among these phenotypic states, isolates sharing the same traits were found at considerable phylogenetic distances. Although most amylase-negative isolates were ST-171 of Clade 1, ST-197 of Clade 2 was also amylase-negative. Similarly, although our urease-positive isolates were confined to the Kurstaki and Sotto lineages of Clade 2, Clade 1 representative ATCC 10987 is known to produce urease [17], and we have encountered a urease producing example of *B. weihenstephanensis* belonging to Clade 3 [18].

Despite the inclusion of multiple isolates representing 15 phenotypic profiles in our sample, 70% of the isolates belonged to either ST-8 (20 isolates), ST-171 (15 isolates), ST-16 (11 isolates) or ST-23 (11 isolates). Respectively, these STs correspond to the classical *Bt* varieties *kurstaki*, *finitimus*, *israelensis* and *morrisoni*. While our approach was not designed to estimate the relative abundance of *Bt* varieties, our results do suggest that these varieties of *Bt*, particularly *Bt israelensis* and *kurstaki*, are very abundant in soil worldwide. The TL phenotype accounts for nearly 25% of our collection, and 7 of 10 TL isolates were ST-16 (*Bt israelensis*). When these isolates are combined with the T, TLE, and TLS isolates that were also found to be ST-16, it can be estimated that this ST may account for nearly 20% of isolates in our collection. An estimate of similar magnitude can be made for the abundance of ST-8. Although we did not select isolates based on crystal morphology, all 15 isolates which we sequence typed that had attached crystals were ST-171 (there were no examples of ST-171 that did not have attached crystals). Since approximately 12% of our collection had attached crystals, it is reasonable to expect that ST-171, and thus *Bt finitimus*, comprises nearly this percentage of the collection. Also highly represented in the sample were ST-241 (6 isolates), and ST-240 (4 isolates). ST-240 represents *Bt toumanoffi*; the original representative of ST-240 was a *toumanoffi* strain (Terrance Leighton and Katie Wheeler; unpublished data), and the sequences of predicted flagellin proteins for ST-240 representative IBL 200 (GenBank accession # EEM97050) and that of the *Bt toumanoffi* serotype strain are identical [19] (GenBank accession # ABD337290). Based on our own collection, we conclude that ST-8 and ST-240 collectively account for the majority of urease-producing *Bt* likely to be encountered in the soil environment. In a previous study, we sequence typed 16 urease-positive isolates from our collection that were not included in the current study (primarily TLU and TLUA phenotypes); among these isolates, 9 were ST-8 and 7 were ST-240 [13]. We have recently shown that among *Bt* toxic to Lepidoptera, only urease-positive isolates were capable of repeated passage through gypsy moth larvae, suggesting that urease production may improve the fitness of *Bt* as a pathogen [20]. Isolates belonging to ST-8 and ST-240 are nearly always toxic to the Lepidoptera, accounting for the correlation between urease production and lepidopteran toxicity we noted previously [11].

The most surprising result of our study, and the best illustration of the lack of correlation between phenotype and phylogeny, was the phenotypic variation that was found within a given ST, or closely related STs. Of the 15 phenotypic profiles represented in our sample, 6 had representatives in ST-8 (TLA, TLE, TLAE, TLU, TLUA and TLUAE). Similarly, among the 6 examples of ST-241 we identified, 5 phenotypes were represented (T, TL, TLSA, TLSAE and TS). Although the majority of amylase-negative isolates were found to represent ST-171, nearly as many ST-171 isolates were amylase-positive. Most isolates associated with the Sotto lineage belonged to ST-16 (11 isolates) and ST-23 (11 isolates) that differ by only two nucleotides in the *purh* allele, and correspond to varieties *israelensis* and *morrisoni*, respectively. A single isolate representing ST-197, an apparent intermediate of ST-16 and ST-23, was also identified. Despite the phylogenetic proximity of these STs, ST-16 isolates typically exhibited a TL phenotype, while TS and TSAE predominated among ST-23, and ST-197 exhibited a “null” phenotype (negative for all traits). Thus, except for being uniformly urease-negative, these highly related STs collectively exhibited all possible individual phenotypic states.

The degree of phenotypic variation that we found among isolates that were genetically indistinguishable by MLSA could be due to changes in gene regulation, or to the loss or gain of genes themselves. Of particular interest to us was the apparent lack of urease production among many examples of ST-8, which includes *Bt kurstaki* and many isolates regarded as pathogens of lepidopteran larvae. In an earlier study, we found that among a group of lepidopteran-toxic *Bt* isolates, the production of urease was very highly correlated with a strain's ability to survive repeated passages in gypsy moth larvae, suggesting that urease was critical to *Bt* living as a pathogen (20). Using primers based on the α-urease gene of ST-8 representative *Bt kurstaki* T03a001 (GenBank accession # ACND01000098), amplicons were obtained from all ST-8 isolates in the current study. All the amplicons had sequences identical to the α-urease gene predicted for T03a001. Thus, differences in urease production among ST-8 isolates in this study appeared due to regulation of the genes, and not their presence or absence. Using the same primers, α-urease was also detected in a ST-240 isolate that did not produce detectable urease activity. These results contrast with those of an earlier study of urease production by *B. cereus* which suggested that expression of urease genes, when present, was constitutive [17].

In summary, phylogenetic position appeared to have very little value in predicting phenotypic characteristics classically used to differentiate *Bt*, with a high degree of phenotypic variation often observed within a particular ST. Nevertheless, in cases where an abundant ST possessed a relatively unusual trait, certain phenotypes were predictive of phylogenetic groups. Despite the diversity of phenotypic profiles included in our selection of isolates, 70% of the isolates belonged to 4 STs that correspond to the classical *Bt* varieties *kurstaki*, *israelensis*, *morrisoni* and *finitimus*.

## Supporting Information

Figure S1 **Maximum likelihood analysis of sequence types 1–594.**
(PDF)

Figure S2 **Maximum likelihood analysis of clade 1 sequence types.**
(PDF)

Figure S3 **Maximum likelihood analysis of clade 2 sequence types.**
(PDF)

Table S1 **Strains utilized in this study, and their properties.**
(PDF)
