# Supplementary figures and images for: Phylogenetic Distribution of Phenotypic Traits in Bacillus thuringiensis Determined by Multilocus Sequence Analysis
Source: PLoS One. 2013 Jun 10;8(6):e66061. doi: 10.1371/journal.pone.0066061 (PMC3677866; doi:10.1371/journal.pone.0066061)

# Supplemental Figure 1

## Maximum Likelihood Analysis of Sequence Types 1 - 594

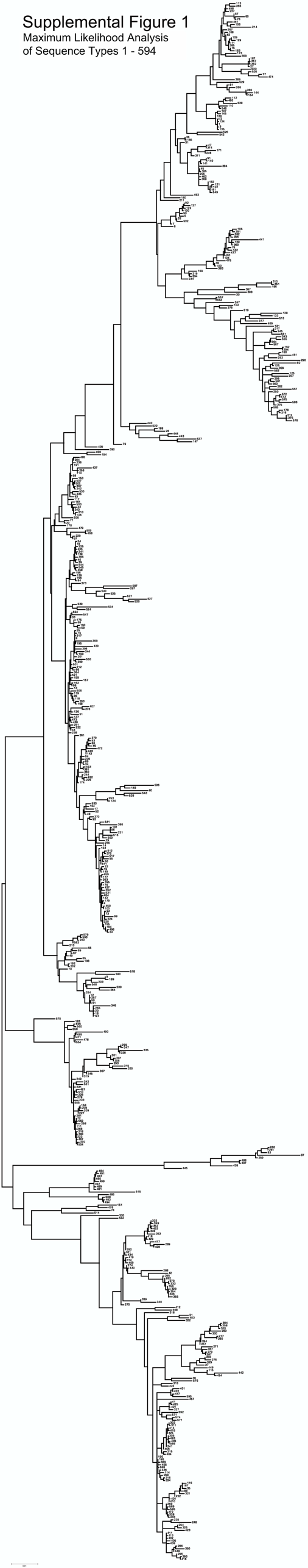

Supplement: Figure S1 — Maximum likelihood analysis of sequence types 1–594. (PDF) [file pone.0066061.s001.pdf]

# Supplemental Figure 2

Maximum Likelihood Analysis  
of Clade 1 Sequence Types

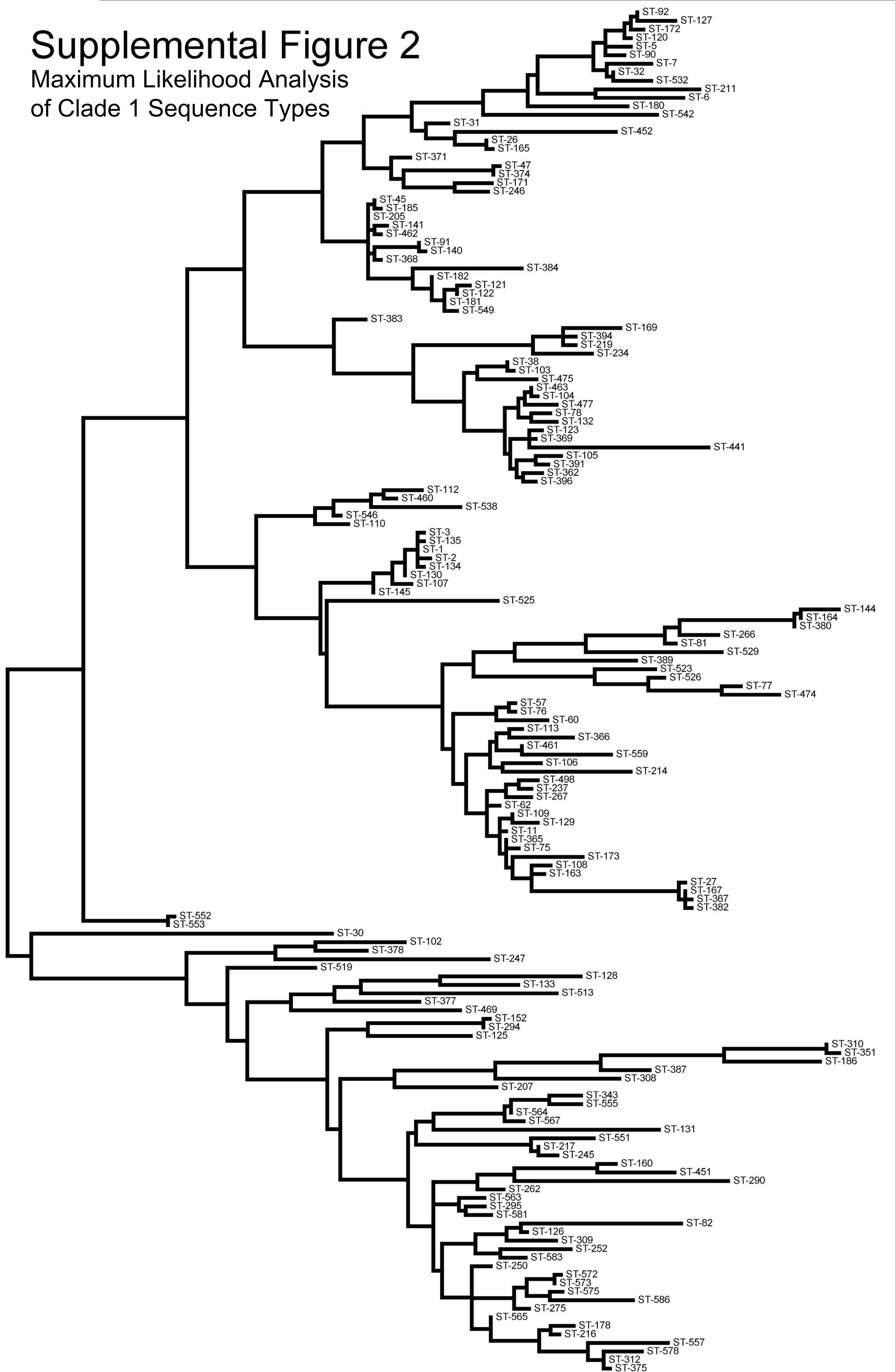

0.005

Supplement: Figure S2 — Maximum likelihood analysis of clade 1 sequence types. (PDF) [file pone.0066061.s002.pdf]

Supplemental  
Figure 3  
Maximum Likelihood  
Analysis of Clade 2  
Sequence Types

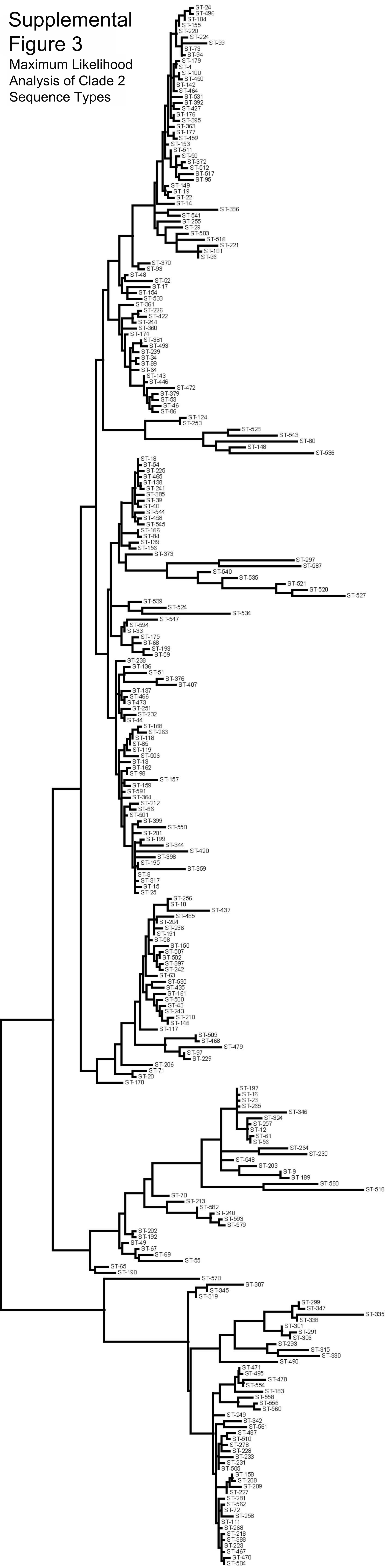

0.005

Supplement: Figure S3 — Maximum likelihood analysis of clade 2 sequence types. (PDF) [file pone.0066061.s003.pdf]
