# Supplementary material for: Phylogenetic Distribution of Phenotypic Traits in Bacillus thuringiensis Determined by Multilocus Sequence Analysis
Source: PLoS One. 2013 Jun 10;8(6):e66061. doi: 10.1371/journal.pone.0066061 (PMC3677866; doi:10.1371/journal.pone.0066061)
Supplement: Table S1 — Strains utilized in this study, and their properties. (PDF) [file pone.0066061.s004.pdf]

Supplemental Table 1. Strains utilized in this study.

Strain	Crystal Type*	Phenotype†	Country	State	Toxicity to Lepid.	Toxicity to Diptera	Seq. Type
IBL 631	attam	LSAE	Morocco		-	-	171
IBL 674	attbp	LSAE	India		-	-	171
IBL 717	attbp	LSAE			-	-	171
IBL 1211	attpyr	LSAE	USA	MD	-	-	171
IBL 3268	attam	LSAE	Nepal		-	-	171
IBL 3568	attam	LSAE	Brazil		-	-	171
IBL 3604	attam	LSAE	Nepal		-	-	171
IBL 3870	attbp	LSAE	USA	WY	-	-	171
IBL 4	am	Ø	USA	NV	-	-	549
IBL 1302	am	Ø	Turkey		-	-	549
IBL 1306	am	Ø	USA	CA	-	+	549
IBL 3476	am	Ø	USA	MD	-	-	197
IBL 77	bp	T	USA	WY	+	-	241
IBL 117	bp	T	USA	WY	+	-	16
IBL 1677	bp	T	USA	WY	+	-	239
IBL 3090	bp	T	USA	NY	+	-	23
IBL 66	am	TL	USA	IL	-	+	548
IBL 189	bp	TL	USA	AR	+	-	240
IBL 4222	am	TL	USA	MD	-	+	16
IBL 429	am	TL	S. Korea		-	+	16
IBL 431	mix	TL	Spain		-	+	16
IBL 439	am	TL	Morocco		-	+	16
IBL 933	bp	TL	Costa Rica		-	-	241

IBL 1374	am	TL	Canada		+	+	16
IBL 3241	am	TL	Norway		-	+	16
IBL 10003	am	TL	Israel		-	+	16
IBL 441	bpcu	TLA	Argentina		-	-	8
IBL 966	bpcu	TLA	Nepal		+	-	8
IBL 1252	bpcu	TLA	S. Korea		+	-	8
IBL 2408	attbp	TLA	Spain		-	-	171
IBL 159	am	TLAE	USA	NM	+	+	8
IBL 1247	am	TLAE	S. Korea		+	-	8
IBL 1254	bpcu	TLAE	S. Korea		+	-	8
IBL 3565	trap	TLAE	USA	MD	-	-	551
IBL 389	bp	TLE	USA	D.C.	+	+	8
IBL 483	bp	TLE	Spain		+	-	593
IBL 621	am	TLE	Egypt		-	+	16
IBL 1268	bpcu	TLE	Argentina		-	+/-	8
IBL 184	am	TLS	USA	DC	-	+	16
IBL 416	am	TLS	China		-	+	16
IBL 520	am	TLS	USA	FL	-	-	547
IBL 1297	am	TLS	USA	MD	-	-	26
IBL 425	bp	TLSA	USA	NY	+	-	10
IBL 480	attam	TLSA	Sweden		-	-	171
IBL 695	bp	TLSA	USA	HA	+	-	22
IBL 3576	bp	TLSA	USA	PA	+	-	241
IBL 299	bp	TLSAE	USA	MD	-	-	241

IBL 464	bp	TLSAE	USA	MD	+/-	-	241
IBL 535	attcry	TLSAE	USA	MD	-	-	171
IBL 1132	attbp	TLSAE	Norway		-	-	171
IBL 1489	attbp	TLSAE	Spain		-	-	171
IBL 1613	attbp	TLSAE	Spain		-	+	171
IBL 3511	am	TLSAE	USA	MD	-	-	592
IBL 3870	attbp	TLSAE	USA	WY	-	-	171
IBL 54	bpcu	TLU	USA	MT	+	-	240
IBL 61	bp	TLU	USA	MT	+	-	240
IBL 563	bpcu	TLU	USA	NC	+	-	8
IBL 1288	bpcu	TLU	Azores		+	-	8
IBL 55	pyr	TLUA	USA	WY	-	+	550
IBL 743	bp	TLUA	USA	MD	+	-	8
IBL 999	bpcu	TLUA	USA	MD	+	+/-	8
IBL 1115	bpcu	TLUA	Iceland	NA	+	-	240
IBL 84	bpam	TLUAE	USA	WI	+	-	8
IBL 88	bp	TLUAE	USA	ND	+	-	8
IBL 455	bp	TLUAE	USA		+	-	8
IBL 533	bp	TLUAE	Vietnam		+	-	8
IBL 661	bp	TLUAE	USA	PA	+	-	8
IBL 749	bpcu	TLUAE	Nepal		+	-	8
IBL 850	bp	TLUAE	Pakistan		+	-	8
IBL 2469	bp	TLUAE	France		+	-	8
IBL 122	bp	TS	USA	WY	+	-	23

IBL 500	bp	TS	Norway		+	-	23
IBL 747	bp	TS	USA	NY	+	-	23
IBL 1410	rect	TS	USA		-	-	23
IBL 1969	bp	TS	India		+	-	23
IBL 3221	bp	TS	Portugal		-	-	241
IBL 3572	bp	TS	USA	NY	+	+	23
IBL 10010	bp	TS			+	-	23
IBL 273	bp	TSAE	USA	WY	+	-	23
IBL 275	bp	TSAE	USA	WY	+	-	23
IBL 3095	am	TSAE	USA	NY	+	-	547
IBL 3579	bp	TSAE	USA	NY	+	-	23

* am-amorphous; att-attached; bp-bipyramidal; cu-cubic; pyr-pyramidal; rect- rectangular; trap-trapezoidal

† Letters indicate positive phenotypic traits: T-amylase; L- lecithinase; U-urease; S-acid from sucrose; A-acid from salicin; E-hydrolysis of esculin
